# Supplementary material for: Dietary Glycemic Index and Load and the Risk of Type 2 Diabetes: Assessment of Causal Relations
Source: Nutrients. 2019 Jun 25;11(6):1436. doi: 10.3390/nu11061436 (PMC6628270; doi:10.3390/nu11061436)
Supplement: Supplementary file 1 [file nutrients-11-01436-s001.pdf]

nutrients MDPI

1. Table S1. Observations in single-sex studies by duration (time, years) of exposure.

Table S1. Observations from the USA in single-sex studies by duration (time, years) of exposure.

Substrate	Study	Follow-up (years)	Risk ratio [a] Mean	(95%CI)	Ref.	Risk relation [b] Mean	(95%CI)
			(Per extreme quintiles)			*(Per 10 units GI)*	
GI	Women	6	1.37	(1.09-1.17)	[1]	1.27	(1.08-1.45)
GI	(NHS I)	24	1.44	(1.33-1.57)	[2]	1.65	(1.48-1.83)
GI		26	1.46	(1.34-1.58)	[3]	1.47	(1.35-1.60)
GI	Women	8	1.59	(1.21-2.10)	[4]	1.50	(1.18-1.90)
GI	(NHS II)	18	1.20	(1.08-1.34)	[2]	1.26	1.10-1.44)
GI	Men	6	1.37	(1.02-1.83)	[5]	1.23	(1.01-1.51)
GI	(HPFS)	22	1.30	(1.15-1.47)	[2]	1.41	(1.20-1.65)
			(Per extreme quintile)			*(Per 80 g GLin 2000 kcal diet)*	
GL	Women	6	1.47	(1.16-1.86)	[1]	1.66	(1.18-2.13)
GL	(NHS I)	24	- [c]	-	[2]	-	-
GL		26	1.32	(1.16-1.51)	[3]	1.84	(0.85-4.00)
GL	Women	8	1.33	(0.92-1.91)	[4]	1.28	(0.78-2.09)
GL	(NHS II)	18	-	-	[2]	-	-
GL	men	6	1.25	(1.09-1.73)	[5]	1.33	(0.88-2.03)
GL	HPFS)	22	-	-	[2]	-	-

a. Risk ratios (point estimates) were from published studies. b. Risk relations (rates) were estimated at present using dose-response meta-analysis. c. All such, fully adjusted model was not presented in the original publication by individual study.

2. Normalisation of fasting blood glucose when lowering the dietary glycemic index in recent medium to long term intervention studies

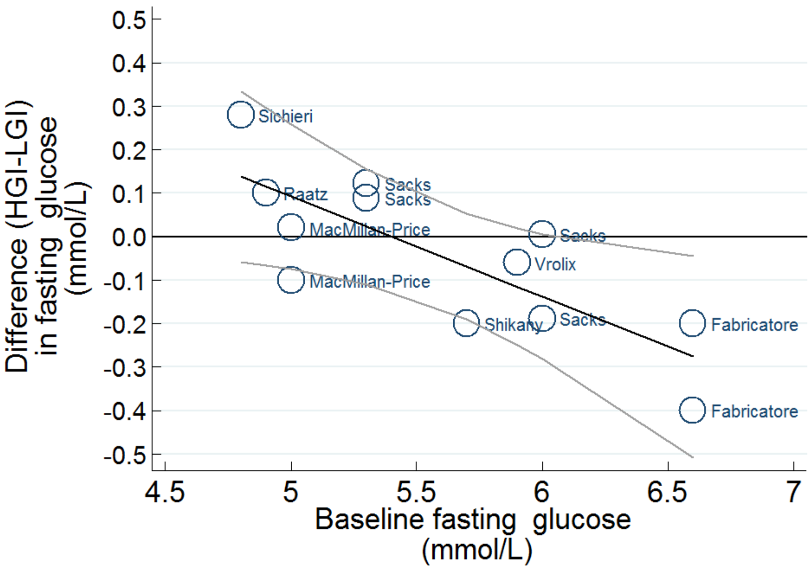

Figure 1. Differences in fasting blood glucose in participants consuming diets of higher and lower glycemic index. Meta-regression curves (grey lines) are 95% confidence intervals. Shown also are the

first author names associated with the studies extracted. Simply throwing these into a forest plot suggest no significant effects but meta-regression shows a significant relation to baseline fasting glucose, considered together with earlier observations [6], there is evidence of lower GI carbohydrate diets reducing both hypoglycaemia and hyperglycaemia among the otherwise random effects.

Following observations in a meta-analyses of human intervention studies published by us elsewhere in 2008 [6] indicating a transition point on the fasting blood glucose concentration for the direction of effect, which was at approx. 5 to 6 mmol/L, the present meta-regression analysis of subsequent findings from randomised control studies in humans (n = 615) from 0.25 to 1.5 years treatment duration [7–13] are confirmatory of the existence of a transition (Figure S1). The slope is the meta-regression relationship based on studies having equal weights with a median effect size SE value of 3.9 mmol/L. The slope was -0.23 mmol/L per mmol/L basal fasting glucose, was significant (P > t = 0.03), and intersected the Y-axis (at y = 0) at a fasting value of 5.4 mmol/L (the transition point) at which the direction effect on fasting glucose changes with baseline fasting blood glucose, and where no effect is expected to be observed.

A weakness of the present meta-regression analysis is the small number of study effects (n = 12), the use of findings from 4 studies at more than one duration of treatment ([12,13], [7,13]), the use of equal weights to alleviate excessive leverage from two studies and heterogeneities in disease states related to blood glucose control and treatment modalities for body weight maintenance and body weight reduction. A strength of the analysis is the agreement with the prior meta-analysis on 50 similar observations [6] over a shorter duration and which showed also a continued greater fall in fasting blood glucose with increasing severity of T2D when lower GI diets were eaten.

Together these analyses indicate both the effect size and direction of effect can be dependent on the effectiveness of blood glucose control in the study groups, and with a transition at about 5.4 mmol/L fasting blood glucose, which otherwise explains heterogeneity in the treatment effect of lower versus higher glycemic index diets. In the present analysis heterogeneity (I^2) was zero after taking account of the status of baseline glucose control. Together with a lack of effect on fasting insulin concentrations by lower versus higher GI diets at below 100 pmol/L [1] these analyses imply a similar implication for the assessment of insulin sensitivity, which is often approximated in models (HOMA IR) by the product of fasting glucose and insulin concentrations and which need robust analytical data [14,15].

A further implication of the meta-analysis (Figure S1 above and in [6]) is that low glycemic diets help to normalise the fasting blood glucose whether it is below a value close to normal or too high. Too few observations are available to verify this at fasting glucose values below 4.5 mmol/L and above 13 mmol/L). Delay in the short term gluco-regulatory mechanisms such as the glucose-fatty-acid cycle [16] and the Staub-Traugott effect likely explain the blood glucose raising potential of lower GI diets at below the transition point.

References

1. Salmeron, J.; Manson, J. E.; Stampfer, M. J.; Colditz, G. A.; Wing, A. L.; Willett, W. C., Dietary fiber, glycemic load, and risk of non-insulin-dependent diabetes mellitus in women. *JAMA* **1997,** *277*, 472-477.
2. Bhupathiraju, S. N.; Tobias, D. K.; Malik, V. S.; Pan, A.; Hruby, A.; Manson, J. E.; Willett, W. C.; Hu, F. B., Glycemic index, glycemic load, and risk of type 2 diabetes: results from 3 large US cohorts and an updated meta-analysis. *Am J Clin Nutr* **2014,** *100*, 218-232.
3. Mekary, R. A.; Rimm, E. B.; Giovannucci, E.; Stampfer, M. J.; Willett, W. C.; Ludwig, D. S.; Hu, F. B., Joint association of glycemic load and alcohol intake with type 2 diabetes incidence in women. *Am J Clin Nutr* **2011,** *94*, 1525-1532.
4. Schulze, M. B.; Liu, S.; Rimm, E. B.; Manson, J. E.; Willett, W. C.; Hu, F. B., Glycemic index, glycemic load, and dietary fiber intake and incidence of type 2 diabetes in younger and middle-aged women. *Am J Clin Nutr* **2004,** *80*, 348-356.

5. Salmeron, J.; Ascherio, A.; Rimm, E. B.; Colditz, G. A.; Spiegelman, D.; Jenkins, D. J.; Stampfer, M. J.; Wing, A. L.; Willett, W. C., Dietary fiber, glycemic load, and risk of NIDDM in men. *Diabetes Care* **1997,** *20*, 545-550.
6. Livesey, G.; Taylor, R.; Hulshof, T.; Howlett, J., Glycemic response and health a systematic review and meta-analysis: relations between dietary glycemic properties and health outcomes. *Am J Clin Nutr.* **2008,** *87*, 258S-268S.
7. Fabricatore, A. N.; Wadden, T. A.; Ebbeling, C. B.; Thomas, J. G.; Stallings, V. A.; Schwartz, S.; Ludwig, D. S., Targeting dietary fat or glycemic load in the treatment of obesity and type 2 diabetes: a randomized controlled trial. *Diabetes Res Clin Pract* **2011,** *92*, 37-45.
8. Sichieri, R.; Moura, A. S.; Genelhu, V.; Hu, F.; Willett, W. C., An 18-mo randomized trial of a low-glycemic-index diet and weight change in Brazilian women. *Am J Clin Nutr* **2007,** *86*, 707-713.
9. Vrolix, R.; Mensink, R. P., Effects of glycemic load on metabolic risk markers in subjects at increased risk of developing metabolic syndrome. *Am J Clin Nutr* **2010,** *92*, 366-374.
10. Shikany, J. M.; Phadke, R. P.; Redden, D. T.; Gower, B. A., Effects of low- and high-glycemic index/glycemic load diets on coronary heart disease risk factors in overweight/obese men. *Metabolism* **2009,** *58*, 1793-1801.
11. Raatz, S. K.; Torkelson, C. J.; Redmon, J. B.; Reck, K. P.; Kwong, C. A.; Swanson, J. E.; Liu, C.; Thomas, W.; Bantle, J. P., Reduced glycemic index and glycemic load diets do not increase the effects of energy restriction on weight loss and insulin sensitivity in obese men and women. *J Nutr* **2005,** *135*, 2387-2391.
12. McMillan-Price, J.; Petocz, P.; Atkinson, F.; O'Neill, K.; Samman, S.; Steinbeck, K.; Caterson, I.; Brand-Miller, J., Comparison of 4 diets of varying glycemic load on weight loss and cardiovascular risk reduction in overweight and obese young adults: a randomized controlled trial. *Arch Intern Med* **2006,** *166*, 1466-1475.
13. Sacks, F. M.; Carey, V. J.; Anderson, C. A.; Miller, E. R., 3rd; Copeland, T.; Charleston, J.; Harshfield, B. J.; Laranjo, N.; McCarron, P.; Swain, J.; White, K.; Yee, K.; Appel, L. J., Effects of High vs Low Glycemic Index of Dietary Carbohydrate on Cardiovascular Disease Risk Factors and Insulin Sensitivity: The OmniCarb Randomized Clinical Trial. *JAMA* **2014,** *312*, 2531-2541.
14. Wallace, T. M.; Levy, J. C.; Matthews, D. R., Use and abuse of HOMA modeling. *Diabetes Care* **2004,** *27*, 1487-1495.
15. Matthews, D. R.; Hosker, J. P.; Rudenski, A. S.; Naylor, B. A.; Treacher, D. F.; Turner, R. C., Homeostasis model assessment: insulin resistance and beta-cell function from fasting plasma glucose and insulin concentrations in man. *Diabetologia* **1985,** *28*, 412-419.
16. Randle, P. J.; Garland, P. B.; Hales, C. N.; Newsholme, E. A., The glucose fatty-acid cycle. Its role in insulin sensitivity and the metabolic disturbances of diabetes mellitus. *Lancet* **1963,** *1*, 785-789.

© 2019 by the authors. Submitted for possible open access publication under the terms and conditions of the Creative Commons Attribution (CC BY) license (http://creativecommons.org/licenses/by/4.0/).
